# Observation of the cancer patient journey: a learning curve for Genetic Counsellors

**DOI:** 10.1186/1897-4287-10-S2-A51

**Published:** 2012-04-12

**Authors:** JA Taylor, KJ Mann

**Affiliations:** 1 Familial Cancer/Adult Genetics, Royal Melbourne Hospital, Parkville, VIC, Australia

## 

Cancer genetic counsellors typically work in hospitals and participate in multidisciplinary clinical teams. However, in their training, genetic counsellors seldom have much exposure to the medical environments in which their clients receive their cancer treatment. Although there is a general understanding about screening, learning a diagnosis, surgical, chemotherapy, and radiotherapy treatments, many of the genetic counsellors have no first hand experience of these processes, making it difficult to comprehend the impact it has on clients.

This project aimed to develop knowledge and awareness of what a cancer patient would experience throughout their cancer journey, therefore increasing the scope for empathy and consideration for clients seen. Secondly, this project aims for genetic counsellors to gain a better appreciation of the risk management procedures and surgeries that enter into the genetic counsellor's discussions with clients of a Familial Cancer Centre (FCC).

Two genetic counsellors approached several senior clinicians (including Medical Oncologists, Gastroenterologists, Surgeons, and Gynaecologists) affiliated with the Royal Melbourne Hospital FCC to observe both consultations and medical procedures in their speciality area.

The genetic counsellors reflected on their observations of the patient's cancer journey and how the patient's experiences may impact on a genetic counselling session. Both counsellors gained a greater insight into the enormity of a cancer diagnosis on an individual.

We wish to share our experience: a greater knowledge of the screening and surgical recommendations that are discussed in a genetic counselling session, an increased awareness of the role of other medical professionals in the patient's cancer journey and a better understanding of how the FCC integrates with the patient's cancer journey. This project warrants extension as we believe that by being better equipped to understand our patients and their needs, we can provide better patient care.

